# The effect of gender, age, and geographical location on the incidence and prevalence of renal replacement therapy in Wales

**DOI:** 10.1186/1471-2369-8-1

**Published:** 2007-01-11

**Authors:** Hugo C van Woerden, Jane Wilkinson, Martin Heaven, Jason Merrifield

**Affiliations:** 1 Department of Epidemiology, Statistics and Public Health, Cardiff University, Heath Park, Cardiff CF14 4XN, UK; 2 National Public Health Service for Wales, South East Region, Temple of Peace and Health, Cathays Park, Cardiff, CF10 3NW, UK; 3 National Public Health Service for Wales, South West Region, 36 Orchard Street, Swansea, SA1 5AQ, UK; 4 Business Services Centre – Mid & West Region (Swansea), The Oldway Centre, 41 High Street, Swansea, SA1 1LT, UK

## Abstract

**Background:**

This study used a cross sectional survey to examine the effect of gender, age, and geographical location on the population prevalence of renal replacement therapy (RRT) provision in Wales.

**Methods:**

Physicians in renal centres in Wales and in adjacent areas of England were asked to undertake a census of patients on renal replacement therapy on 30 June 2004 using an agreed protocol. Data were collated and analysed in anonymous form.

**Results:**

2434 patients were on RRT in Wales at the census date. Median age of patients on RRT was 56 years, peritoneal dialysis 58 years, haemodialysis 66 years and transplantation 50 years. The three treatment modalities had significantly different age-specific peak prevalence rates and distributions. RRT age-specific prevalence rates peaked at around 70 years (1790 pmp), transplantation at around 60 years (924 pmp), haemodialysis at around 80 years (1080 pmp) and peritoneal dialysis did not have a clear peak prevalence rate. Age-specific incidence of RRT peaked at a rate of 488 pmp at 79 years, as did incidence rates for haemodialysis, which peaked at the same age. Age had less effect on the initiation of peritoneal dialysis, which had a broad plateau between the early fifties and late seventies. Kidney transplantation rates were highest in the early fifties but were markedly absent in old age.

**Conclusion:**

Differences in the provision of RRT are evident, particularly in the very elderly, where the gender difference for haemodialysis is particularly marked. The study illustrates that grouping patients over 75 years into a single age-band may mask significant diversity within this age group. Significant numbers of very elderly patients who are currently not receiving RRT may wish to receive RRT as the elderly population increases, and as technology improves survival and quality of life on RRT.

The study suggests that if technologies that are more effective were developed, and which had a lower impact on quality of life, there might be up to a 17% increase in demand for RRT in those aged over 75 years; around 90% of this increased demand would be for haemodialysis.

## Background

Since the introduction of renal replacement therapy (RRT) in the UK in 1946 [1], its use has risen steadily. As survival has improved, there has been an increasing willingness to offer dialysis to a wider range of patients including older patients. This has resulted in rising annual incidence (acceptance) and prevalence rates. In the 1960s, dialysis was rarely offered to patients over 65 years[2]. The acceptance rate for RRT has steadily risen from around 22 per million population (pmp) [3] in the UK population in 1982, to 133 pmp in Wales in 2003 [4]. The prevalence rate has also risen, in England and Wales, from around 393 pmp [5] in 1993 to over 700 pmp [4] in 2003. The continuing rise in demand for RRT has imposed a huge strain on resources in renal services [6]. There have been recurrent demands for yet more investment[7] and some surprise that demand has not reached a plateau. Geographical boundaries in Wales have changed and there was concern to ensure that further investment addressed, rather than increased, inequalities in gender, age and geographical location. Some concern had also been expressed regarding the reliability and independence of available data. This study attempted to address and explain some of the issues behind the ongoing requirement for further investment.

A number of factors underlie the rising demand in RRT. Technological advances have encouraged the offering of RRT to older patients with greater co-morbidity. Technological advances have also improved survival, increasing the incidence and prevalence of patients on RRT. A rapidly aging population, has, and will increasingly, exacerbate the effect of the above factors, as population predictions suggest that the number of people aged over 60 years will rise by around 65 per cent over the next 50 years. Similarly, the number of people aged over 75 years is expected to double, and the number of people aged 90 years and over is expected to more than triple [8].

Separate analysis of data on RRT prevalence or incidence in the very elderly has historically been scarce and the use of summary measures of RRT, encompassing wide age bands, may also have obscured the underlying shifts in the numerators and denominators in the elderly. This study has consequently used age-specific rates, which have a number of advantages over age-standardised rates as they are unaffected by differences in demography [9]. This allows the separating out of age related factors and factors associated with changes in the provision (demand and supply) of RRT. Age-standardised rates can only be compared with other age-standardised rates using the same age-standardised population. For example, age-standardised rates for the UK population cannot be compared with age-standardised rates for the European population or the North American population. In contrast, age specific rates can be compared between any two populations without being affected by different population pyramids in the different populations [10,11].

Age-specific rates have advantages over age-standardised rates, particularly for the oldest age band in any analysis. For example, two identical age-standardised rates for individuals "over 75 years" could represent two very different populations. One population where all the population in this age-band were aged 75–80 years (i.e. had died by the age of 80 years) and another population where there were a large proportion of patients living on to 90–100 years and over. Detailed differences within the age-band "over 75 years" are very relevant to the cost of providing RRT, given the demographic shift occurring in the western world. The cost of a patient aged 100 years on RRT is likely to differ from the cost of treating a patient aged 75 years because of general frailty and co-morbidity.

A doubling of the number of patients on RRT over, say 80 years or over 90 years will have a major impact on service provision as these patients are frail and have multiple co-morbidities. However, because the very elderly make up only a small proportion of the population, such a rise in RRT would only result in a small rise in the overall age-standardised RRT rate for "the over 75 years". The additional workload for the service is consequently masked. The similar generation of age-specific rates in other countries or populations would facilitate debate on the emerging question as to the true level of unmet need for RRT in the ninth and tenth decades of life.

Other potential inequalities in RRT provision are also important to consider. This study has therefore examined the effect of gender, geographical location and modality of RRT on RRT prevalence rates. Insufficient numbers were available to assess the effect of these factors on incidence rates.

## Methods

A census of all patients in Wales on RRT was undertaken on 30 June 2004. This date was chosen, as it is the middle day of the year and population projections are calculated for that day of the year. A protocol was developed with input from renal physicians and circulated to previously agreed contacts in all renal centres in Wales and those in neighbouring areas in England used to treat Welsh patients. Data gathered included modality of RRT on that date, postcode, date of birth, gender and an indicator of whether the patient had first started RRT on or after 1 July 2003.

To comply with data protection legislation, no names or other personal identifiers were collected. The data were cleaned by comparing all postcodes with a database of all current and past postcodes in Wales and then removing patients with non-Welsh postcodes. Patients with missing or invalid postcodes or dates of birth were checked with the provider Trust. Data sets were combined in a secure Microsoft Sequel Server 2000 database. Duplicate entries were identified as those where the date of birth, gender and postcode were identical. The number of deceased patients in the collated data set was estimated to ensure that "ghost" patients would not unduly inflate our RRT rates.

The study was conducted in conformity with the requirements of the Declaration of Helsinki. Advice was sought from relevant experts to ensure compliance with Data Protection requirements. The South Wales Renal Managed Clinical Network determined that formal ethical approval was not required, as identifiable data was only made available to Health Solutions Wales staff, who have authorisation from the Patient Information Advisory Group (PIAG) to handle Patient Episode Database Wales (PEDW) data under Section 60 of the Health and Social Care Act 2001. This view was supported by Health Commission Wales, an executive agency of the Welsh Assembly Government, who commissioned the study.

To clean the data set further, possible entries representing deceased patients were identified by comparing date of birth, gender and postcode in our data set against the Welsh NHS Administrative Register (AR) held by Health Solution Wales. This comparison was made separately for patients treated by Welsh NHS Trusts and for one English Trust where there was concern that there might be a significant number of deceased patients. However, entries potentially representing deceased patients could not be removed from the dataset as the matching process could suggest, but not confirm, that a particular matching entry might represent a deceased patient. Our methodology was designed to estimate the potential size of this problem without having access to NHS numbers and to determine whether the numbers involved were sufficiently small as to be ignored in our calculation of RRT rates.

Denominator data were obtained from mid-year estimates of the population of Wales in 2004 (by Local Health Board) calculated by the Office of National Statistics for each one-year age band from the age of one to 89 years. For the one-year age bands between 90 and 99 years data from the 2001 census was used, as mid-year estimates are not available for ages over 89 years. No patients with an age of over 100 years were identified in the database and RRT rates for 100 years and over were consequently set to zero. Rates (pmp) were calculated for each one-year age band for the three modalities of RRT, i.e. haemodialysis, peritoneal dialysis and transplantation.

Moving averages of 3, 5, 7, 9, 11, 13 and 15 years were explored using an equal number of years above and below the age in question. An 11-year moving average was identified as providing the best balance between retention of definition of changing features of the graph, minimising lags in the peaks and troughs in the graph, and yet smoothing spikes in the data. Data for some graphs were truncated below 18 yrs and above 90 years because of numerators of fewer than 5 cases in some of the individual one-year age bands.

To indicate the probability of being on a given form of RRT at any given age, the proportion of patients on each of the three modalities of RRT was calculated for each one-year age band and converted into a percentage so that the three percentages came to 100%. An 11-year moving average was also applied to this data as described above. All data was analysed and graphed using Microsoft Excel™.

Postcodes were converted into a grid reference and recorded as an Easting and a Northing. Grid references were then mapped to Local Health Board (LHB) areas in Wales. This allowed the calculation of acceptance and prevalence rates by LHB. 95% confidence intervals were calculated for these rates using a SQL procedure which performs the confidence interval algorithm described by Newcombe[12] and Wilson [13].

## Results

Summary measures for the data are shown in Table 1. There were 63.2% males and 36.8% females, a male to female RRT ratio of 1.7:1. The median age for patients on RRT was 56 years, peritoneal dialysis 58 years, haemodialysis 66 years and transplantation 50 years.

**Table 1:** Summary measures for patients on RRT in Wales

	RRT	Peritoneal Dialysis	Haemodialysis	Transplantation
Number of patients	2434	299	813	1322
Median age (years)	56	58	66	50
Mean age (years)	54.70	56.60	62.81	49.44
Standard deviation	16.29	16.51	15.97	14.31

The result of our extensive data cleaning and validation exercise was as follows. The database contained 2434 records with unique dates of birth, which could also be fully matched to current or historical Welsh postcodes. An additional 167 records could not be matched to a Welsh LA area; 110 of these records clearly represented English postcodes; an additional 35 were probable English postcodes, and 22 records had invalid postcodes, which could potentially have been Welsh postcodes. The maximum number of missing Welsh records in our analysis would therefore be <1% (22/2434). In conclusion, the 167 unmatched and consequently excluded records identified during data validation largely represented English and not Welsh patients and their exclusion would consequently not have affected our analysis.

Comparison with the Welsh NHS AR indicated that only 10 records (0.45%) represented deceased patients that had not been removed from the renal registers in various NHS Trusts. A handful of patients may have been double counted as a result of errors in dates of birth or postcode changes. Detailed analysis also suggested that a handful of errors might also have occurred because patients moved house during the months preceding the census. Once again, our data validation indicated that these issues did not significantly affect our data.

A significant issue was identified in relation to one English NHS Trust. Comparison between NHS AR data and data on patients provided with a transplant by this Trust suggested around 3% of the patients still on the trust's renal register were deceased. These patients were removed from our database and consequently would not have affected our results. However, our data validation exercise indicated that other data collection exercises in the UK may have potentially significant errors due to inadequate removal of deceased patients from some trust renal registers.

**Figure 1:**
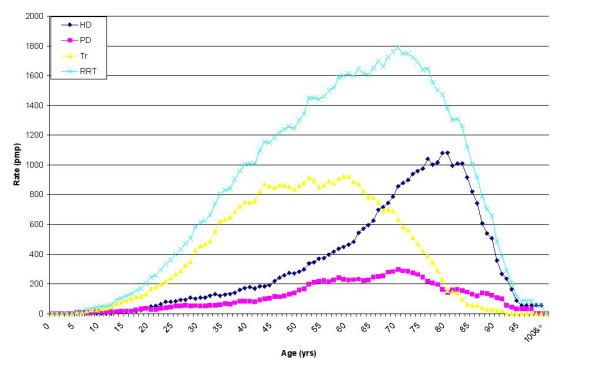
Age-specific prevalence rates (moving average pmp) of RRT for the renal stock in Wales on 30 June 2004.

Age-specific prevalence rates for the three modalities of RRT had significantly different peak rates and distributions. RRT age-specific prevalence rates (Figure 1) peaked at around 70 years with a rate of around 1790 pmp; haemodialysis age-specific prevalence rates peaked at around 80 years with a rate of around 1080 pmp; peritoneal dialysis rates did not have a clear peak; and transplantation age-specific prevalence rates peaked at around 60 years with a rate of around 924 pmp.

**Figure 2:**
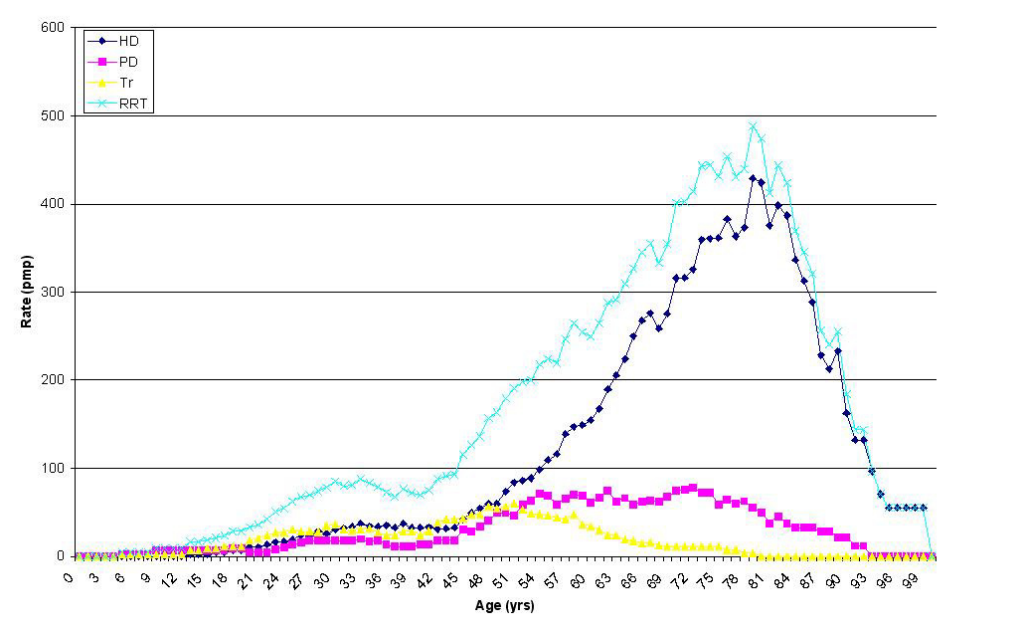
Age-specific incidence rates (moving average pmp) of renal patients *alive at the census date *and starting RRT in the year prior to 30 June 2004 in Wales.

Age-specific incidence rates for RRT (Figure 2) peaked at a rate of around 488 pmp at 79 years. This was very heavily influenced by the high incidence rates for haemodialysis, which peaked at the same age. Age appears to have much less effect on initiation of peritoneal dialysis, which had a broad plateau between the early fifties and late seventies. Rates of new transplantation were highest in the mid-fifties, but were markedly absent in old age. This is in contrast to the renal transplant prevalence rates, which indicates that a number of patients in old age have functioning transplanted kidneys. These patients appear to have received their transplants at a much younger age.

**Figure 3:**
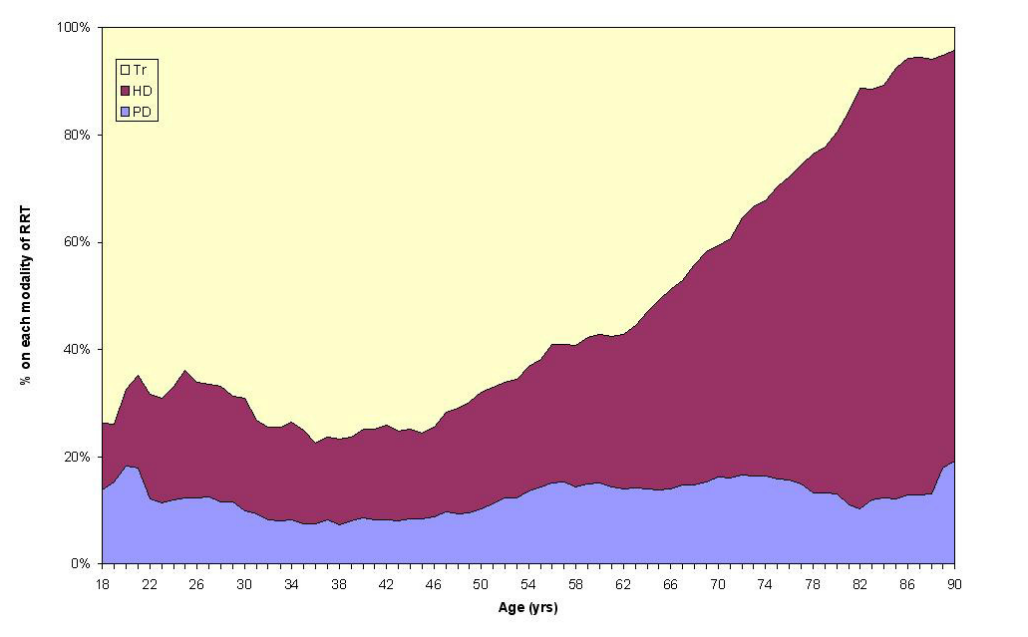
Age-specific probability (moving average pmp) of being on a given modality of RRT for the renal stock in Wales on 30 June 2004.

Figure 3 explores the relative probability of being on a given modality of RRT at any given age. The graph indicates that the probability of being on peritoneal dialysis remains at around 10–15% for all age groups. Until the age of 60 years the probability of being on haemodialysis remains at around 25%, after which it rises steeply and over the age of 80 years an individual on RRT had around an 80% probability of being on haemodialysis. In contrast, the probability of having a transplant falls steadily from the age of 45 years.

**Figure 4:**
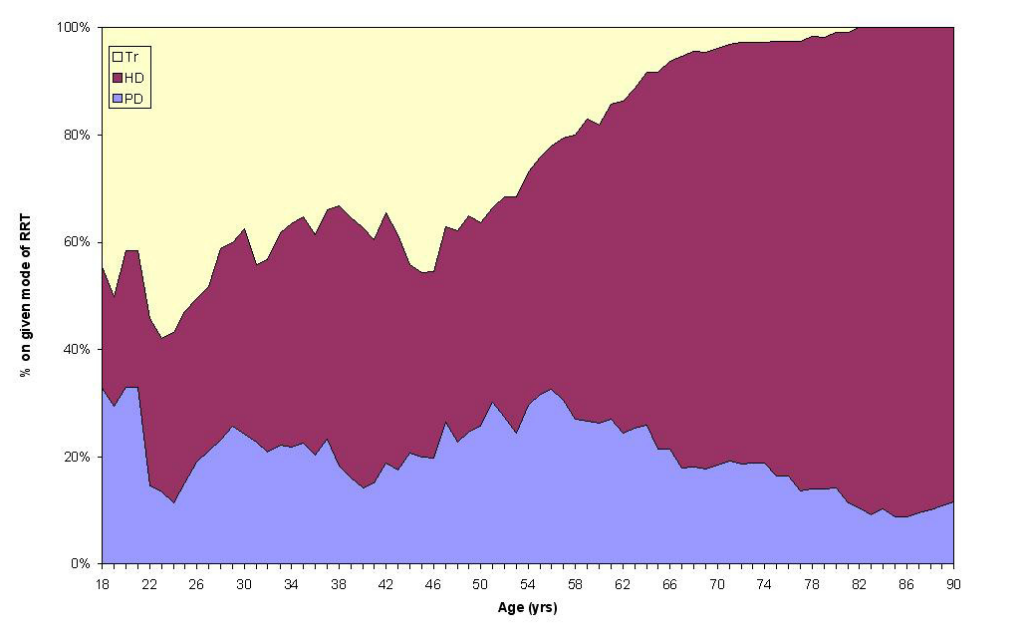
Age-specific probability (moving average pmp) of renal patients *alive at the census date *having started a given modality of RRT in the year before 30 June 2004 in Wales.

Figure 4 indicates the probability, based on an individual's age, of commencing a specified modality of RRT when RRT is first initiated. The graph suggests that physicians prefer giving younger patients renal transplantation or starting them on peritoneal dialysis. With increasing age, and particularly after the mid-fifties, physicians appear to have an increasing preference for starting patients on haemodialysis. However, some physicians seem to consider transplantation for occasional patients up until their late sixties, and some physicians started patients on dialysis into their late eighties. The extent to which patients have contributed to these treatment decisions is unclear.

**Figure 5:**
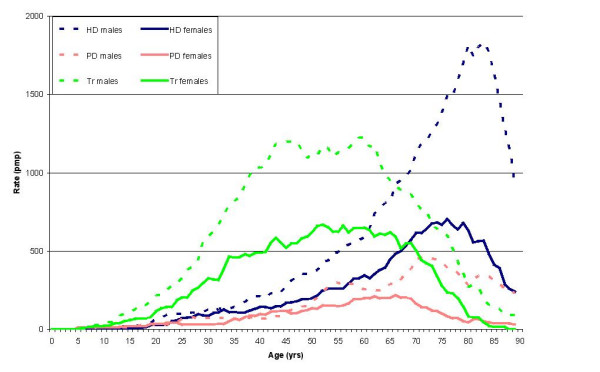
Age-specific prevalence rates (moving average pmp) of RRT for the renal stock in Wales on 30 June 2004 for males and females.

The effect of gender with age is examined in Figure 5. The graph indicates that at every age, men receive rates of RRT that are higher, or at least as high, as the rates for women. This gender difference applies to all three modalities of RRT. The difference is most marked for transplantation in middle age and for haemodialysis in old age.

**Figure 6:**
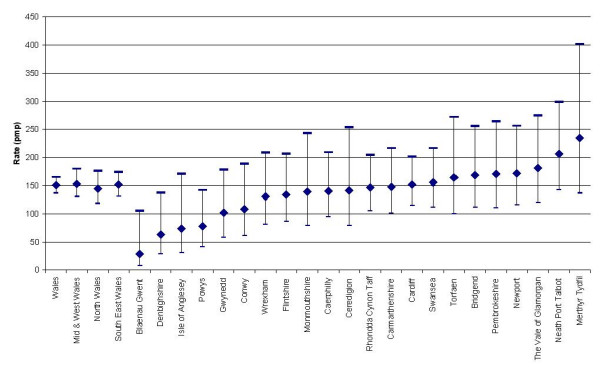
Acceptance rates for different LHBs in Wales in the year preceding 30 June 2004.

**Figure 7:**
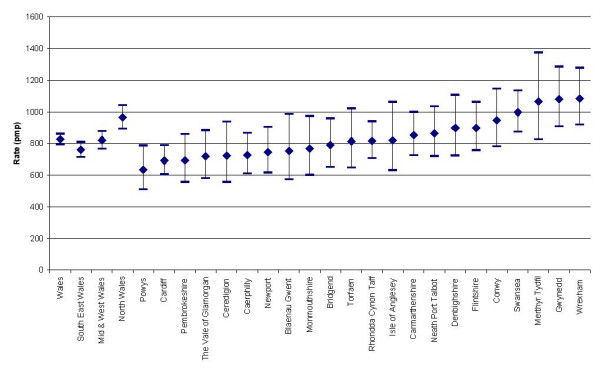
Prevalence rates for different LHBs in Wales in the year preceding 30 June 2004.

Geographical location also has an effect on RRT rates of incidence (acceptance) and prevalence (Figures 6 and 7). North Wales, Swansea, Gwynedd and Wrexham area rates appear to be higher than the all Wales rates. Cardiff and Powys area rates appear to be lower than the all Wales rates. There is wide variation in the acceptance rate, ranging from under 50 pmp to over 200 pmp.

**Figure 8:**
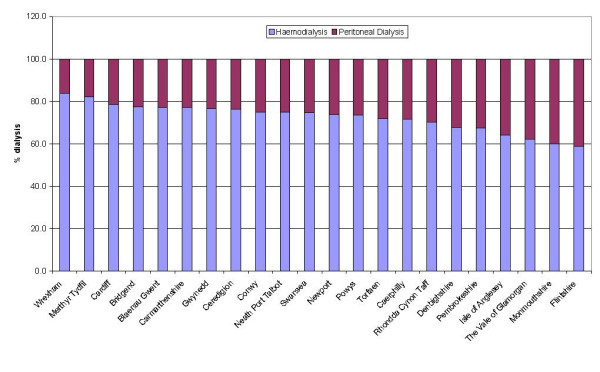
Proportion of patients in different LHBs on peritoneal and haemodialysis in Wales on 30 June 2004.

Geographical location also influences the relative proportion of patients on peritoneal or haemodialysis (Figure 8). The proportion of patients on haemodialysis in the LHB areas at the extremes of Figure 8 (Wrexham and Flintshire) is not statistically significantly different from the proportion of patients on haemodialysis in Wales as a whole (73.1%). However, there is a 25.1% (95%CI 8.0–40.6%) difference between the proportions of patients on haemodialysis between these two areas.

## Discussion

This study demonstrates significant variation in the provision of RRT by gender, age, and geographical location. Differences in RRT rates were most marked by gender and age, and to a lesser extent by geographical location. Some of the gender difference in prevalence may be due to risk factors shared by cardiovascular disease and end stage renal failure, both of which are commoner in men. However, some of the gender differences in the very elderly, particularly the differences in haemodialysis, may be indicative of underlying inequality in treatment provision.

Implications for service provision can be drawn from the findings. Age-specific prevalence for peritoneal dialysis was relatively similar at all ages, and consequently, peritoneal dialysis services need to be designed to serve all age groups in the population. In contrast, support services for patients with renal transplants needs to be focused on those under 65 years. For example, attendance at outpatient clinics may significantly interfere with working age patients who have a transplant.

Patients in Wales under the age of 50 years have a greater than 50% chance of having a transplant (Figure 3), which is encouraging for younger patients waiting for a transplant.

The probability of being on peritoneal or haemodialysis varies depending on the LHB in Wales. This may represent the preferences of different clinicians for particular treatment modalities, and a wide range of socio-economic determinants, as well as random chance.

There are a number of possible reasons why the tertiary English NHS Trusts, which treat Welsh transplant patients, might have a higher number of patients recorded as being alive, when the NHS Administrative Register suggested that they were deceased. Firstly, there may be less accurate recording of death in this Trust. However, tertiary care centres may see patients less frequently, if joint care is undertaken with a locally based clinician. Consequently, if a patient dies in a local hospital, the tertiary centre may not be aware of the patient's demise. Our findings suggest that there is merit in linking databases held by different Trusts, in forming links between Trust databases, national renal databases and the NHS AR using patients NHS numbers. This would reduce inaccuracies around deceased patients, patients who have recently moved address, and patients attending multiple renal centres.

This study suggests that there may be a significant number of untreated elderly patients with end stage renal failure. Figure 1 indicates that the rate of RRT provision rises with age but falls after the mid-seventies. This is unexpected given the rising trend in morbidity up to this age. The true underlying rate of end stage renal failure is very unlikely to fall over the age of 75 years and this suggests that there may be a gap between need and supply in this very elderly age group. The discrepancy may be occurring because clinicians and patients are taking into account the personal, physical, social and psychological costs of treatment, and the very poor survival curves associated with current treatment over this age, and decide not to offer or accept RRT.

We can provide a crude estimate of the number of potentially untreated patients over the age of 75 years by linear extrapolation of the population rate for RRT from 1790 pmp at 75 years to 2500 pmp for the age group 100 years. Similarly, we can extrapolate the haemodialysis rate from 1000 pmp at 85 years to 1500 pmp at 100 years. Extrapolating in this way would suggest that an additional 442 individuals over the age of 75 years might wish to be treated, if RRT methods were developed that provided excellent rates of survival and low negative impact on patients' quality of life. This unmet "need" would represent a 17% increase on current demand for RRT.

These figures provide a crude estimate of the possible increase in future demand in the very elderly, without taking into account other factors such as demographic shift in the population, or increased provision of RRT to those under 75 years. In summary, these findings suggest that, as RRT technologies continue to improve, an increasing number of very elderly patients are likely to present for treatment.

Patients over the age of 75 years have a very high probability of being on haemodialysis. Population predictions for the next decade for Wales suggest that this population group will expand the most. Unless the current treatment pattern changes significantly, the high use of haemodialysis in old age (Figures 4 &5) combined with the demographic bulge predicted in this age group suggests that the demand for haemodialysis in the very elderly will continue to rise at a higher rate than in younger age groups.

### Limitations of this study

Our study has a number of limitations. We have used a different method of calculating incidence from that used by the renal registry, which includes patients who started RRT in the year before the census date, but who also died in that year. Our figures would, therefore, be expected to indicate a lower rate than if these patients were included. Our incidence figures would have been improved if our data collection had included patients who died in the preceding year. Our data is cross-sectional: longitudinal data gives better estimates of incidence, although cross sectional studies such as ours, which ask for changes over the proceeding year, can be used.

A renal physician from every renal centre in Wales, and NHS Trusts in England that treat Welsh patients, was used as a point of contact. Data managers in each trust were also involved. As each trust keeps a database of patients receiving treatment for ESRF we were confident that all individuals receiving treatment had been identified. However, not all patients with ESRF present for treatment and not all patients with ESRF are offered or receive treatment. Prevalence estimates of ESRF based on treatment databases are consequently underestimates of the true prevalence.

Based on our discussion with renal physicians in Wales we are confident that we have identified all patients receiving treatment by a renal physician. However, very low acceptance rates reported in some areas (under 50 pmp) could theoretically be the result of local treatment and failure to refer by non-renal physicians.

Data on ethnicity was not collected in this study, as there are relatively low rates of non-white populations in most of Wales. However, this would have provided some useful additional information as end stage renal failure is affected by ethnic origin.

The peak rate for a given modality, shown in the graphs using a moving average, is around two to four years younger than the true value. This is because the moving average brings forward the peak in the graphs by between two to four years when calculated using a 'window' moving from left to right.

Co-morbidity was not assessed in this study; however, it is an important factor in the elderly. There is some evidence that the presence and severity of co-morbid conditions is more important than age as a predictor of survival after commencement of dialysis[14,15]. Further information on the range, variation, severity and relative influence of co-morbidity on survival would have been informative.

Conservative options have also not been addressed in this study, but are important to a proportion of patients with end stage renal failure. There is significant potential for improved prevention and earlier conservative treatment to slow deterioration in renal function and affect the number of patients requiring RRT [16]. A decision not to offer treatment may also be appropriate [15] although age alone may sometimes inappropriately be used as a criterion for withholding treatment. In one study, the decision to withhold dialysis increased at a rate of 12% for every 10 years of increasing age even after adjustment for conditions such as dementia and/or dependency [17].

### Comparison with other studies

Most of the significant epidemiological work on renal disease in the UK has been undertaken by the UK Renal Registry [4] and its Scottish counterpart [18]. The annual report of the UK Renal Registry in particular contains information submitted by all the renal units in Wales and has expanded year on year. The negative influence of geographical distance from a renal unit has also previously been demonstrated in Wales [19]. In Scotland, the effect of social class [20], co-morbidity [21] and referral patterns of GPs and non-nephrologists [22] have been studied in detail. Co-morbidity and non-referral are often linked to age [21,23] but the epidemiological consequences for very elderly patients has rarely been examined [24].

## Conclusion

The study explores the importance of providing RRT rates for patients over 75 years to more accurately reflect the rapid changes in RRT provision that are occurring in this age group. The study identifies variation, and potential inequality, in the provision of RRT by age, gender and geographical location. The gender difference in haemodialysis rates in the very elderly is particularly marked and emphasises the value of calculating age and gender specific rates of RRT rather than using one combined age-standardised rate to represent all those over 75 years.

Significant numbers of very elderly patients who are currently not receiving RRT may wish to have this as the elderly population increases, and as technology improves survival and quality of life on RRT. The study also suggests that if technologies that are more effective were developed, which had a lower impact on quality of life, there might be up to a 17% increase in demand for RRT in those aged over 75 years, and given current patterns of treatment, around 90% of this increase in demand would be for haemodialysis.

## Competing interests

The author(s) declare that they have no competing interests.

## Authors' contributions

JW, MH and HvW designed the study, and collated the results; MH and JM analysed the data using a SQL database; HvW drafted the paper, which was then approved by all the authors.

## Pre-publication history

The pre-publication history for this paper can be accessed here:


